# Neurophysiological Basis of Multi-Scale Entropy of Brain Complexity and Its Relationship With Functional Connectivity

**DOI:** 10.3389/fnins.2018.00352

**Published:** 2018-05-29

**Authors:** Danny J. J. Wang, Kay Jann, Chang Fan, Yang Qiao, Yu-Feng Zang, Hanbing Lu, Yihong Yang

**Affiliations:** ^1^Laboratory of FMRI Technology, Stevens Neuroimaging and Informatics Institute, Keck School of Medicine, University of Southern California, Los Angeles, CA, United States; ^2^Department of Psychology, Center for Cognition and Brain Disorders, Hangzhou Normal University, Hangzhou, China; ^3^Neuroimaging Research Branch, National Institute on Drug Abuse, National Institutes of Health, Baltimore, MD, United States

**Keywords:** multiscale entropy (MSE), complexity, BOLD fMRI, electrophysiology, functional connectivity (FC)

## Abstract

Recently, non-linear statistical measures such as multi-scale entropy (MSE) have been introduced as indices of the complexity of electrophysiology and fMRI time-series across multiple time scales. In this work, we investigated the neurophysiological underpinnings of complexity (MSE) of electrophysiology and fMRI signals and their relations to functional connectivity (FC). MSE and FC analyses were performed on simulated data using neural mass model based brain network model with the Brain Dynamics Toolbox, on animal models with concurrent recording of fMRI and electrophysiology in conjunction with pharmacological manipulations, and on resting-state fMRI data from the Human Connectome Project. Our results show that the complexity of regional electrophysiology and fMRI signals is positively correlated with network FC. The associations between MSE and FC are dependent on the temporal scales or frequencies, with higher associations between MSE and FC at lower temporal frequencies. Our results from theoretical modeling, animal experiment and human fMRI indicate that (1) Regional neural complexity and network FC may be two related aspects of brain's information processing: the more complex regional neural activity, the higher FC this region has with other brain regions; (2) MSE at high and low frequencies may represent local and distributed information processing across brain regions. Based on literature and our data, we propose that the complexity of regional neural signals may serve as an index of the brain's capacity of information processing—increased complexity may indicate greater transition or exploration between different states of brain networks, thereby a greater propensity for information processing.

## Background

### Neural complexity

Complexity is a key feature characterizing the behavior of physiological systems of a living organism (Lipsitz, 2004). The brain, an information processing system with 10–100 billion neurons and ~10^14^ synapses, exhibits the highest degree of complexity among all organs in the human body. In recent years, interest in understanding the dynamics of neural signals and their relation to information processing has increased steadily (Garrett et al., 2013). Neural complexity can be framed as the range, or capacity, of the brain to explore alternative states (Honey et al., 2007; Ghosh et al., 2008; Shew et al., 2009; Friston et al., 2012). Regions of the human brain, indeed systems of neurons, are known to organize transiently into functionally-connected networks for brief periods—from tenths of a second to seconds—only to become reorganized moments later as elements of networks with different functions. These dynamics are readily made visible using EEG (Tucker et al., 1986; Bullmore and Sporns, 2009; Betzel et al., 2012), fMRI (Allen et al., 2014; Barttfeld et al., 2015), among other measurement tools, when coupled with time series analytic methods such as independent components analysis (ICA) (Bell and Sejnowski, 1995; Beckmann and Smith, 2004; Smith et al., 2012). This flexibility of rapid transition implies not only a low energy barrier between states, but also a relatively wide repertoire of quasi-stable states that can self-organize rapidly. The brain's fluid movement among different states has been conceptualized by Friston et al. (2012), who argue that a characteristic feature of the brain is its tendency to wander, or not settle into any particular state. It is posited that systems engaging in greater transition or exploration between different states (i.e., a higher level of complexity) have greater potential and propensity for information processing (McDonough and Nashiro, 2014).

An important parameter defining complex or chaotic systems is the self-similar or “fractal” behavior across multiple measurement scales, and the tendency of the frequency spectra showing an inverse power-law (1/*f*^*n*^–like) scaling pattern. Scale-free activity is present at almost every temporal and spatial scale in the brain (He et al., 2010); it has been observed in neuronal spike trains (Gisiger, 2001; Takahashi et al., 2004), neurotransmitter release (Lowen et al., 1997), spontaneous local field potential (LFP) (Leopold et al., 2003; Milstein et al., 2009), electrocorticography (ECoG), resting state fMRI (rs-fMRI) (Zarahn et al., 1997; Wang et al., 2003; Bullmore et al., 2004), and in fluctuations of human cognitive and behavioral performance (Gilden, 2001). For instance, neuronal populations exhibit a type of activity termed *neuronal avalanches*, characterized by the occurrence of bursts of activity that, despite their wide variation in sizes and durations, still follow precise statistical properties according to a power law (Plenz and Thiagarajan, 2007; Petermann et al., 2009; Ribeiro et al., 2010).

**Figure 1:**
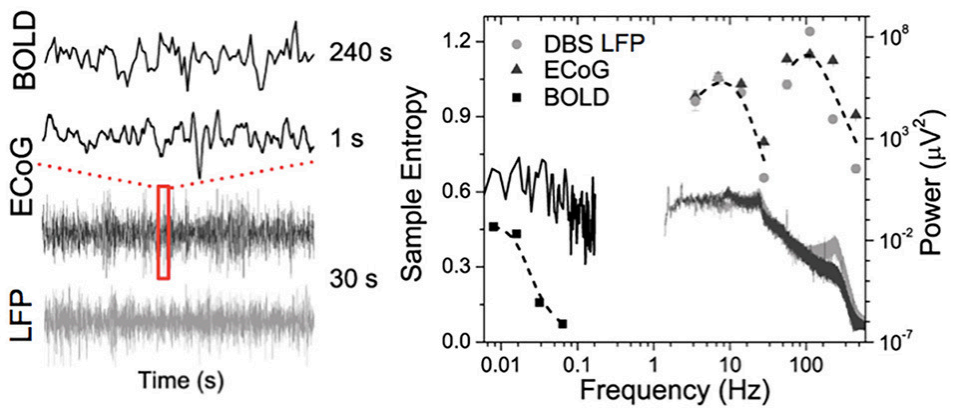
Time courses (left) and corresponding power spectra (solid lines) and entropy (symbols) results of BOLD fMRI from GPi and GPi LFP and primary motor ECoG signals in a PD patient.

Figure 1 shows data from our lab using wavelet based entropy analysis (Smith et al., 2015) of rs-fMRI data from the globus pallidus internus (GPi), GPi LFP, and primary motor ECoG signals recorded during surgical implementation of deep brain stimulation (DBS) in a patient with Parkinson's disease (PD). All modalities exhibit a general power-law behavior in their power spectrum. Entropy across all modalities shows similar behavior with increasing trends toward low frequencies. In addition, similar “small world” topological structure of brain networks has been observed from micro- and meso-scopic circuits to large scale brain networks (Bassett and Bullmore, 2006; Valverde et al., 2015) as well as in EEG microstate sequences (Van De Ville et al., 2010). The nearly ubiquitous power-law behavior suggests that the brain operates near states of self-organizing criticality (SOC), providing many desirable features in optimizing the brain's computational capabilities including sensory input processing, information transfer, and storage (Bak et al., 1987; Plenz and Thiagarajan, 2007; Ribeiro et al., 2010). The fact that such scale-free spatial and temporal pattern “replicates” itself across different modalities and measurement scales also offers a unique opportunity to bridge cellular and circuit level recordings with systems level brain imaging—a major goal of the BRAIN initiative (Alivisatos et al., 2012).

### Quantification of neural complexity

People have been interested in understanding and characterizing self-organizing systems since the 1950s, and have developed basic principles like center manifold theorem (Carr, 1981) and synergistic treatment of high-dimensional self-organizing systems, such as the brain (Ginzburg and Landau, 1950), as well as the slaving principle (Haken, 1983) to highlight the role of endogenous fluctuations. These fluctuations model the dynamics attributable to fast (stable) modes that become enslaved by the slow (unstable) modes, which determine the macroscopic behavior. The time constants of these macroscopic dynamics are necessarily greater (or slower) than those of the underlying microscopic dynamics. Importantly, these endogenous fluctuations follow the scale free (power-law) distribution (Friston et al., 2011).

During the past few decades, a variety of measures derived from the fields of nonlinear statistics and information theory have been developed to describe the dynamics of physiological systems (Goldberger, 1996). Nonlinear dynamic analysis using fractal dimension (*FD*) and Hurst exponent (*H*) can be used to quantify the complexity of biological signals (Natarajan et al., 2004; Di Ieva et al., 2015). The complexity of real-world time series of finite length, however, cannot usually be estimated with reasonable precision. For the analysis of such typically short, and noisy, time series Pincus introduced approximate entropy (ApEn) as a family of non-linear statistics to quantify regularity in physiological finite length time series (Pincus, 1991). ApEn, and its variants (e.g., sample entropy or SampEn) (Richman and Moorman, 2000), measure the conditional probability that runs of patterns that are similar for *m* contiguous observations remain close on subsequent incremental comparisons (*m*+*1*). Higher ApEn values indicate generally that the process is less predictable (or more complex). Subsequently, multi-scale entropy (MSE) analysis (Costa et al., 2002) was developed to more accurately differentiate complex processes from random fluctuations, by calculating the entropy of a signal at multiple time scales. In MSE analyses, a series of entropy values are calculated on coarse-grained time series that are constructed by averaging the original time series over a range of scales. Systems with 1/*f* power spectra exhibit constant entropy over various time scales (due to their fractal properties), whereas random noise shows a marked decrease in entropy at longer time scales (as random fluctuations are smoothed out). To date, ApEn, SampEn, and MSE have been applied successfully to biological signals such as cardiac electric activity (ECG), blood pressure, respiratory patterns, hormonal release, electromyogram (EMG), and brain electric activity (EEG), to distinguish healthy function from disease, and to predict the onset of adverse health-related events (Kaplan et al., 1991; Pincus and Keefe, 1992; Ryan et al., 1994; Schuckers and Raphisak, 1999; Abásolo et al., 2005; Pincus, 2006; Szaflarski et al., 2012; Takahashi, 2013).

**Figure 2:**
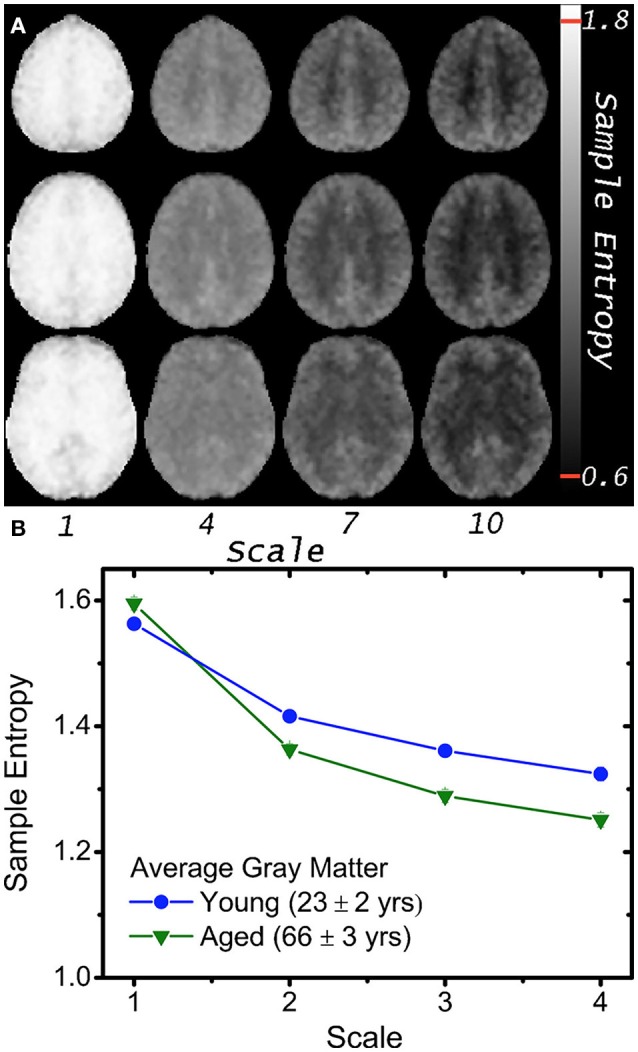
**(A)** Mean MSE images of 5 volunteers at scales 1, 4, 7, and 10; note greater gray and white matter contrast in higher MSE scales (i.e., lower temporal frequencies). **(B)** Average gray matter MSE for 8 young and 8 aged volunteers at 4 scales with greater age differences at higher scales (*p* < 10^−4^). Plotted error bars are four standard errors of the respective means, and approximately the size of the symbols (With permission from Smith et al., 2014).

Functional MRI based on the blood oxygen level-dependent (BOLD) contrast (Ogawa et al., 1990; Kwong et al., 1992) is one of the most widely used methods for noninvasive monitoring of the temporal dynamics of brain physiology (e.g., cerebral blood flow) and neuronal activity (see review Cohen and Bookheimer, 1994). Functional connectivity (FC) analysis has revealed that multiple regions of the brain, even structurally distant, are employed in parallel during both task and rest conditions (Biswal et al., 1995; Raichle et al., 2001; Damoiseaux et al., 2006). Recent fMRI and electrophysiological studies suggest that FC may exhibit dynamic changes within time scales of seconds to minutes (i.e., non-stationary processes; Chang and Glover, 2010). These non-stationary properties may not be captured fully by linear statistical methods such as cross-correlation analysis. We (Liu et al., 2013; Smith et al., 2014, 2015) and others (Yang et al., 2013; Wang et al., 2014) have recently explored the use of entropy measures as indices of the complexity and regularity of BOLD fMRI time-series in healthy young and elderly populations (see Figure 2) as well as in subjects associated with genetic risks of dementia, subjects with ADHD (Sokunbi et al., 2013) and schizophrenia (Takahashi et al., 2010). Significant correlations between complexity measures and functional connectivity across brain networks have also been reported (McDonough and Nashiro, 2014). These emerging studies support the validity of using entropy measures of rs-fMRI to characterize the spontaneous fluctuations of brain physiology and neuronal activities non-invasively at systems level.

### Relationship between neural complexity and FC

Although initial data showed promising results, the neurophysiological basis of complexity (MSE) of electrophysiology and fMRI signals as well as their relations to functional connectivity (FC) remain unclear. The complexity of fluctuating neural activity has been linked to the probability of neuronal firing, and to the likelihood of synchrony between brain regions. It has been postulated that more predictable signals (less neural complexity) facilitate phase relationships between brain regions, thus increasing the probability of synchrony, and information exchange across distributed brain regions. In contrast, the opposite is expected with more irregular signals (greater neural complexity; Ghanbari et al., 2015). The degree of synchrony across brain regions may also differ between the fine and coarse time scales that are associated with different levels of neural complexity (McDonough and Nashiro, 2014). Coarse time scales may reflect long-range interactions across distributed neural populations, while fine time scales may reflect interconnectivity among local neural populations (Vakorin et al., 2011; McIntosh et al., 2014). The purpose of this conceptual analysis paper is to investigate neurophysiological basis of complexity (MSE) of electrophysiology and fMRI signals, and to test the hypotheses on the relations between complexity and FC through the following perspectives: (1) theoretical simulations of network dynamics with the neural mass model (NMM) based brain network modeling, (2) animal models with concurrent recordings of fMRI and electrophysiology data in conjunction with pharmacological manipulations, and (3) MSE and FC analyses of rs-fMRI data with high spatiotemporal resolutions acquired with multiband echo-planar imaging (EPI) sequences of the Human Connectome Project (Feinberg et al., 2010; Moeller et al., 2010).

## Theoretical modeling

### Large-scale brain network models

Theoretical modeling has unique strength for understanding and potentially predicting the complex behavior of brain networks under different experimental or behavioral conditions. Since the Hodgkin–Huxley model developed in the 1950s to explain the causes of single neuron spikes, biophysical models for large-scale brain activity have been developed to understand perception and behavior, as well as the determinants of large-scale neuroimaging data. As summarized by a recent review (Breakspear, 2017), there are primarily two types of models to describe collective dynamics of neuronal ensemble (e.g., a cortical column), including the Fokker–Planck equation (FPE) that assumes uncorrelated neuronal activities within an ensemble; and NMM that assumes strong coherence of neuronal activities within an ensemble which is biologically more meaningful (Breakspear, 2017).

As description of a local population of interacting neurons, NMMs can be integrated into mesoscopic circuits, and macroscopic systems to form so called “ensemble of ensembles”—large-scale brain network models (BNMs). BNMs integrate NMMs with research findings of complex brain networks (Jirsa et al., 2010; Mejias et al., 2016), since dynamics within each NMM results from both local population activity and influences of other NMMs, and here the coupling of NMMs is informed by anatomical connectivity such as the primate CoCoMac and diffusion MRI-based data (i.e., structure-functional model). This feature makes BNMs a favorable tool in simulation studies aimed to understand and interpret resting-state fMRI data. Indeed, existing modeling work in primates combined a static skeleton of structural connectivity with regional neural dynamics, signal transmission delays, and noise to understand the emerging properties of large-scale brain networks (Honey et al., 2007, 2009; Ghosh et al., 2008; Deco et al., 2009).

### Simulation of neural complexity and FC

In this work, the Brain Dynamics Toolbox (https://github.com/breakspear/bdtoolkit) was used for simulation that includes NMM based BNMs (Heitmann and Breakspear, 2017). The NMM describes local populations of densely interconnected inhibitory and excitatory neurons whose behaviors are determined by voltage- and ligand-gated membrane channels. Sodium and calcium channels display a nonlinear sigmoid-shaped graph of voltage-dependent conductance. Potassium channel conductance is modeled in a more complex manner, exponentially relaxing toward its voltage-dependent state. A medium-scale (mesoscopic) array (BNM) is then constructed from these local nonlinear populations by introducing long-range pyramidal connections, mimicking glutamate-induced synaptic currents. Spatiotemporal patterns arise through reentrant excitatory–excitatory feedback (Breakspear et al., 2003). Activity in the system arises purely from nonlinear instabilities (and noise can also be added). Oscillations are hence spontaneous and self-organizing.

We used CoCoMac (Honey et al., 2007) as structural connectivity matrix and set all physiologically measurable parameters within their accepted ranges to generate dynamically plausible behavior (Breakspear et al., 2003), while ensuring different nodes wouldn't stay synchronized because of too strong coupling. For each node, a simulated spike train with 10,000 data points was generated, and coarse-grained time series were constructed by averaging the original time series over scales of 2–400, respectively. MSE at each scale was calculated as the Sample Entropy (SampEn) of the corresponding time series, defined as the log likelihood of *m*+1-length patterns matching within a tolerance threshold *r*, provided they were matching for the first *m* points (Richman and Moorman, 2000; Smith et al., 2014). We used pattern length *m* = 3 and pattern matching threshold *r* = 0.2 for simulated data with a length of 10,000 data points and low noise. The mean MSE was then generated across the full scale as well as across 5 frequency bands (delta, theta, alpha, beta, and gamma), respectively.

**Figure 3:**
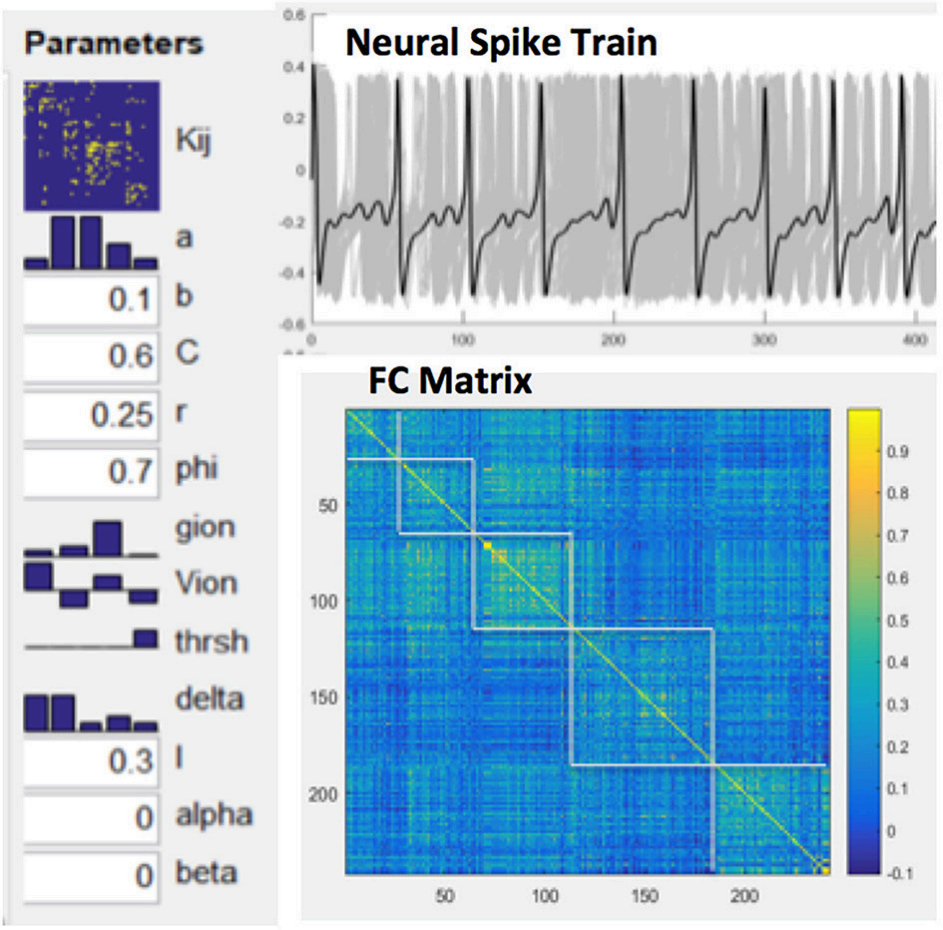
Model parameters for NMM based brain network modeling, simulated neural spike train of one node (solid curve) and rest nodes (gray curves) and resultant FC matrix with 5 modules.

Functional connectivity (FC) was computed by Pearson correlations between spike train time series of all pairs of network nodes. The relationship between MSE and FC of nodal spike trains was investigated by repeated simulations (total 50) while randomly varying excitatory-to-excitatory connectivity (Aee) which represents the strength of long-range FC. For each simulation we randomly picked an Aee value between 0 and 0.55 to generate spike trains using the BNM. A random noise with additive volatility of 0.001 was included in the spike train time course. Cross-correlations between the mean MSE and FC measures were calculated to estimate their associations across the whole CoCoMac and its 5 modules, respectively (see below). Figure 3 shows our modeling parameters and simulated neural spike trains and FC matrix based on the CoCoMac (Honey et al., 2007). The simulated neural spike train of one node (solid curve) exhibits a pattern of synchronization—desynchronization with the rest nodes (gray curves). The FC matrix clearly demonstrates 5 modules of functionally connected networks, which is highly consistent with the partition of CoCoMac into 5 structural modules (corresponding to frontal/orbitofrontal, inferior temporal, frontal/superior temporal, prefrontal/motor/somatosensory, and occipital/visual/prefrontal regions; Harriger et al., 2012).

**Figure 4:**
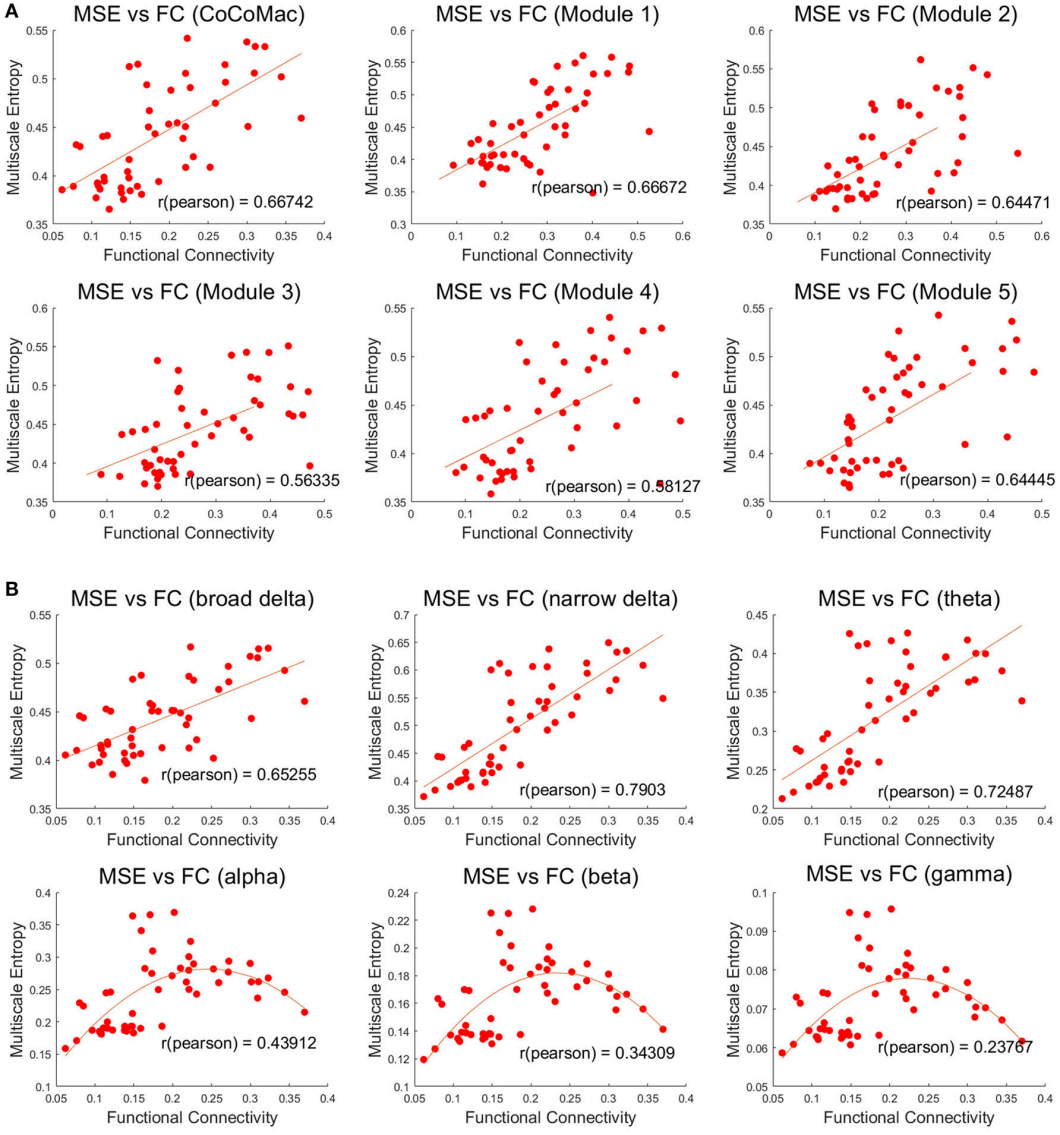
**(A)** Positive correlations between mean MSE and FC of whole CoCoMac and 5 modules (*p* < 0.001). MSE was averaged across the time scale of 1 to 400 corresponding to temporal frequency of 0.5–200 Hz. **(B)** Associations between frequency band dependent MSE and FC of the whole CoCoMac. MSE was averaged within the delta (broad 0.5–4 Hz and narrow 2.7–4 Hz), theta (4–7 Hz), alpha (8–15 Hz), beta (16–31 Hz), gamma (32–200 Hz). Each data point (total 50) was generated with mean MSE and FC values simulated with a random excitatory-to-excitatory connectivity (Aee) value (0–0.55) as well as adding random noise. Fitted linear and quadratic functions are also plotted in each sub-figure.

Both MSE and FC increase with larger Aee in the whole CoCoMac as well as its 5 modules (*r* ≥ 0.78, *p* ≤ 0.001, Figures S1, S2). As a result, significant positive correlations (*r* ≥ 0.56, *p* ≤ 0.001) between FC and MSE were observed in the whole CoCoMac as well as its 5 modules by randomly varying Aee (Figure 4A). In the above analyses assuming a sampling rate of 200 Hz, MSE was averaged across the time scale of 1–400 corresponding to temporal frequency of 0.5–200 Hz. We also calculated averaged MSE of different frequency bands (delta, theta, alpha, beta, gamma) and the results are displayed in Figure 4B. There is a trend of decreasing associations between MSE and FC from the theta (4–7 Hz), alpha (8–15 Hz), beta (16–31 Hz), to gamma (32–200 Hz) band. In fact, for alpha, beta and gamma bands there is an inverted U-shaped relationship between MSE and FC, which is best fit by a quadratic function (see Figure 4B). For the delta band, we calculated MSE over the full (0.5–4 Hz) and a narrower range (2.7–4 Hz) to match the time points for averaging of the rest frequency bands, and the narrower band of high delta frequencies showed the highest correlation between MSE and FC (*r* = 0.79) among all frequency bands.

In addition, we performed simulations without added random noise. As expected, the MSE values became smaller and the FC larger. Nevertheless, the increasing trend of MSE and FC with larger Aee as well as their positive correlations remain unchanged.

## Animal experiment with concurrent electrophysiology and MRI

For this section, we reanalyzed concurrent electrophysiology and MRI data acquired in rats at 9.4T (Jaime et al., 2017). The purpose of the original study was to understand the neurophysiological basis of spontaneous rs-fMRI fluctuations and FC through cross-frequency phase-amplitude coupling (PAC) between concurrently recorded local field potential (LFP) and BOLD signals in the striatum at resting state and by agonizing the AMPA (α-amino-3-hydroxy-5-methyl-4-isoxazolepropionic acid) receptors within the ventral tegmental area (VTA). All experimental procedures were approved by the NIDA-IRP Animal Care and Use Committee. Silicon-based MRI-compatible microelectrode arrays (NeuroNexus) were implanted into the left striatum in rats (*N* = 8), and a microinjection cannula was implanted above the VTA for AMPA microinjections to modulate VTA neuronal activity and connected striatum areas. After 1-week recovery from surgery, rats underwent repeated concurrent fMRI and electrophysiological recording experiments on a Bruker 9.4T scanner. A single shot GE-EPI sequence (*TR* = 1,500 ms, *TE* = 15 ms, matrix = 64 × 64, FOV = 1.92 × 1.92 cm^2^, 5 × 0.3 mm slices) was used to acquire BOLD data for ~60 min (7.5 min per epoch). MR gradient and RF induced artifacts on LFP were corrected as described in Jaime et al. (2017). The timing of each artifact segment was identified based on concurrently acquired slice trigger signal from the scanner. Each 60 ms LFP segment immediately following the trigger signal had high-amplitude (up to ±5 V) fast changing signals. These segments were replaced by linear interpolation. The linearly interpolated segments were then replaced by cubic-spline interpolation of the data from 35 ms before and 35 ms after the artifact segments. Finally, LFP data were low-pass filtered to 100 Hz and down-sampled to 50 Hz for final MSE analysis. Repeated fMRI/LFP data were recorded pre and post microinjection of AMPA (1 μl, 100 μM) in VTA. MSE was calculated for both LFP and fMRI data (pattern length *m* = 2, matching threshold *r* = 0.5, scale = 1–40 for LFP and 1–10 for BOLD time series). FC of fMRI data was calculated using ventral striatum as the seed area. ANOVA was then applied to detect brain regions with significant mean MSE and/or FC changes by AMPA injection. Cross-correlations were calculated between mean MSE of LFP and fMRI in ventral striatum as well as FC of fMRI within the ventral striatum cluster showing significant FC changes following AMPA injection.

**Figure 5:**
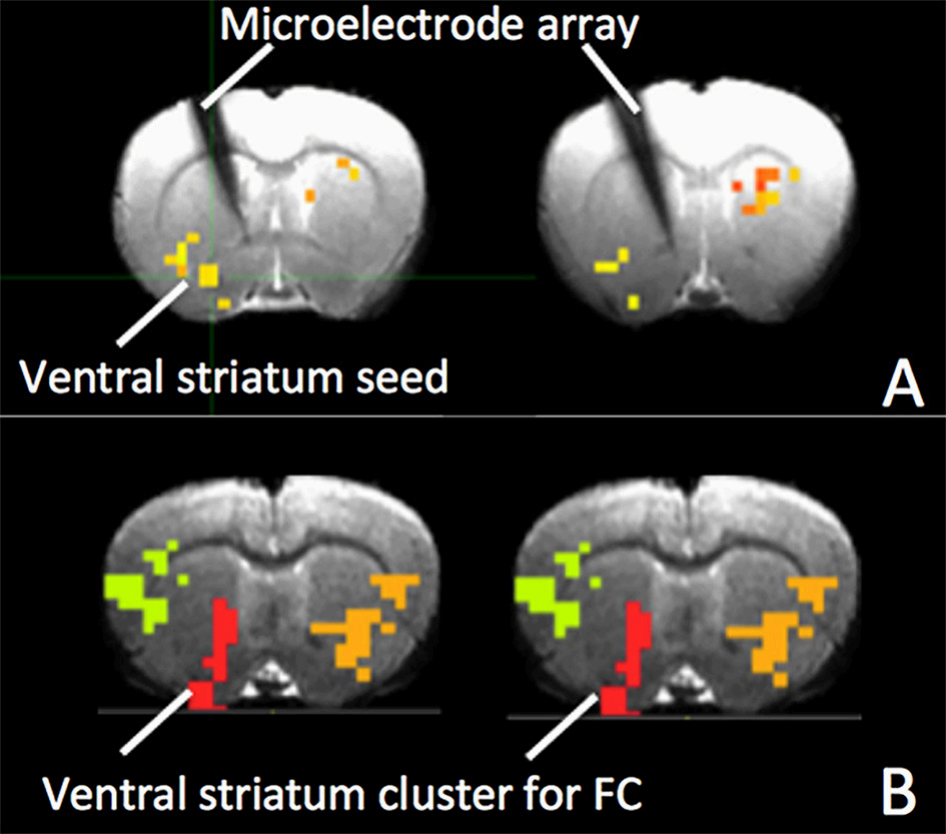
**(A)** The electrode and clusters with significant fMRI MSE changes due to AMPA injection are shown on gradient echo (GRE) images of rat brain. The cluster in the ventral striatum is used as seed for FC; **(B)** Clusters with significant FC changes in response to VTA AMPA injection. The ventral striatum cluster (red) was used for calculation of mean FC.

**Figure 6:**
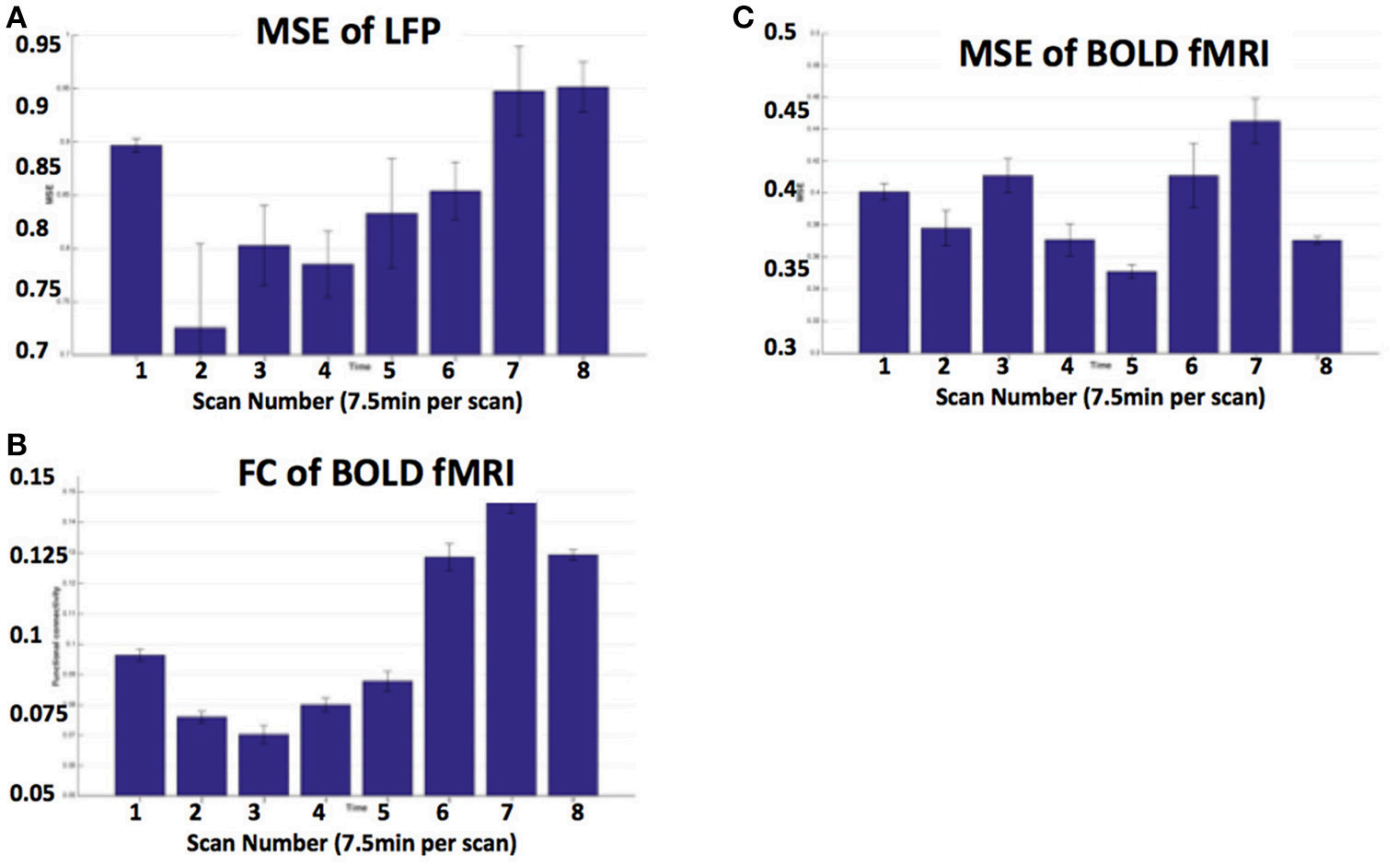
**(A)** MSE of LFP recorded at electrode tip (close to ventral striatum); **(B)** BOLD FC using ventral striatum as a seed; **(C)** MSE of BOLD fMRI (0.04–0.07 Hz) in ventral striatum following VTA AMPA injection in 8 animals. Each scan is 7.5 min and scan 1 is baseline. FC is averaged within the ventral striatum cluster shown in Figure 5B.

Figures 5A,B show MRI of electrode positions and clusters with significant mean MSE and FC changes due to AMPA injection in animal experiment. As shown in Figures 6A,B, both mean MSE of LFP (recorded at electrode tip) and FC of BOLD fMRI (within ventral striatum cluster) decrease following AMPA injection, with a significant correlation (*r* = 0.505, *p* < 0.001) between the two measures (calculated with partial correlation controlling effect of animals). All metrics return to baseline at 37.5 min post AMPA with overshoot afterwards. We also calculated MSE of different frequency band of LFP and the results are listed in Table 1. Consistent with simulation results shown in Figure 4B, there is a trend of decreasing associations between MSE and FC from the theta, alpha, beta, to gamma band. In addition, MSE of BOLD fMRI (0.04–0.07 Hz) in ventral striatum shows a trend of decreasing followed by signal recovery in response to AMPA injection (Figure 6C), which is significantly correlated with that of fMRI FC (*r* = 0.43, *p* = 0.0005).

**Table 1:** Correlations between MSE of different frequency band of LFP and FC of BOLD fMRI signals (calculated with partial correlations controlling for effect of animals).

**Frequency band (Hz)**	***R*-value**	***P*-value**
Delta (1.25–4)	0.454	<0.001
Theta (4–7)	0.56	<0.001
Alpha (7–13)	0.545	<0.001
Beta (13–25)	0.505	<0.001
Gamma (25–50)	0.444	<0.001

## Human FMRI with HCP SMS EPI sequences

We analyzed resting state fMRI (rs-fMRI) data of 20 subjects from the HCP database (Van Essen et al., 2013). These participants were unrelated to each other, relatively healthy individuals that were free of a prior history of significant psychiatric or neurological illnesses, but could have a history of smoking, heavy drinking, or recreational drug use without having experienced severe symptoms. All participants gave written informed consent as approved by the Washington University in St. Louis institutional review board.

Data were acquired at 3T with TR/TE = 720/33 ms, multiband-factor 8, FA = 52°, gradient-echo EPI readout and 2 mm isotropic resolution (Smith et al., 2013a). For each subject two sessions of rs-fMRI were analyzed with phase encoding from left to right (LR) and right to left (RL), respectively. Datasets were preprocessed according to HCP minimal preprocessing pipeline (Glasser et al., 2013). Additionally we regressed out physiological noise (white matter and cerebrospinal fluid signal fluctuations calculated as average signal fluctuations within eroded tissue probability maps) and motion parameters (3 translations and 3 rotations as well as their first derivatives). Preprocessed data was submitted to a group independent component analysis (GIFT toolbox; Calhoun et al., 2001) and four components were selected, which represent the Default Mode Network (DMN), Left and Right Executive Control Networks (L-/RECN), and the Salience Network (SAL).

For all nodes within these networks we computed FC between all nodes using conventional Pearson correlations. Second, MSE was calculated for 40 temporal scales (pattern length *m* = 2, matching threshold *r* = 0.5; McDonough and Nashiro, 2014; Smith et al., 2014). This allowed comparing FC to signal complexity at different temporal frequencies. Results from the LR and RL phase encoded rs-fMRI sessions were averaged for each subjects and the following tests were performed: (i) To test for potential relations between the local signal dynamics (MSE) and network coherence (FC) we correlated the overall network FC with overall network MSE across scales to identify a global association between network connectivity and network complexity. The overall network measures were computed by means of averaging across all node-to-node connections for FC and all nodes' MSE, respectively; (ii) We correlated MSE to the average FC of each node to identify the frequency range where MSE correlates to nodal connectivity.

**Figure 7:**
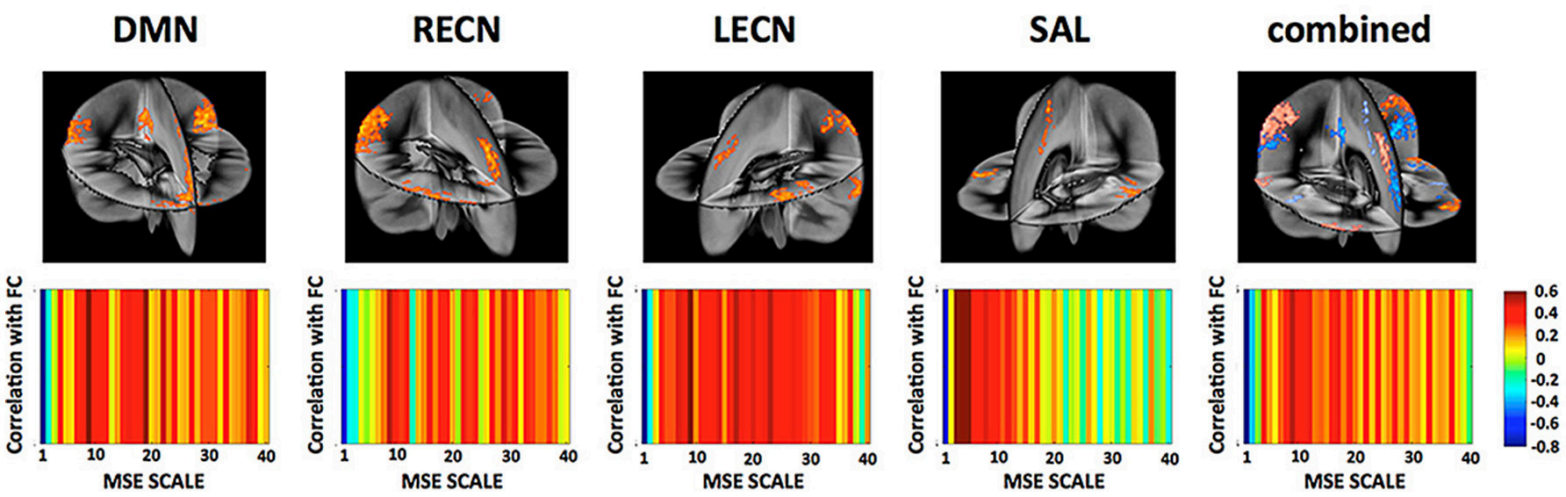
**(Top)** Networks and their nodes as identified from group ICA displayed in a three dimensional rendering of the brain. [DMN, Default Mode Network; LECN, left executive control network; RECN, right executive control network]. **(Bottom)** Correlation between a network global FC and their MSE across all frequency scales. While network FC shows negative correlations between MSE and FC at higher frequencies (fine scales) there is indication that this relationship reverses at mid- to low-frequencies. This is in line with the view that MSE at high frequencies represents more local processing independent from other nodes whereas MSE at lower frequencies represents the information transfer between distributed nodes.

On the network level, we found that the overall network FC is inversely related to the overall network MSE at higher temporal frequencies (0.347–0.694 Hz) across subjects while positively correlated to MSE at lower temporal frequencies (0.020–0.087 Hz; Figure 7). This result was found to be consistent for all four networks representing higher cognitive functions as well as when combining all nodes of all networks into a whole-brain network. This finding is consistent with previous reports (McDonough and Nashiro, 2014) and the theory that higher-frequency oscillations originate from smaller local neuronal populations, whereas low-frequency oscillations encompass larger long-range neuronal populations. Hence while MSE at higher frequencies represents local processing the association between FC and low frequency MSE represents the information transfer between distributed nodes of the network.

**Figure 8:**
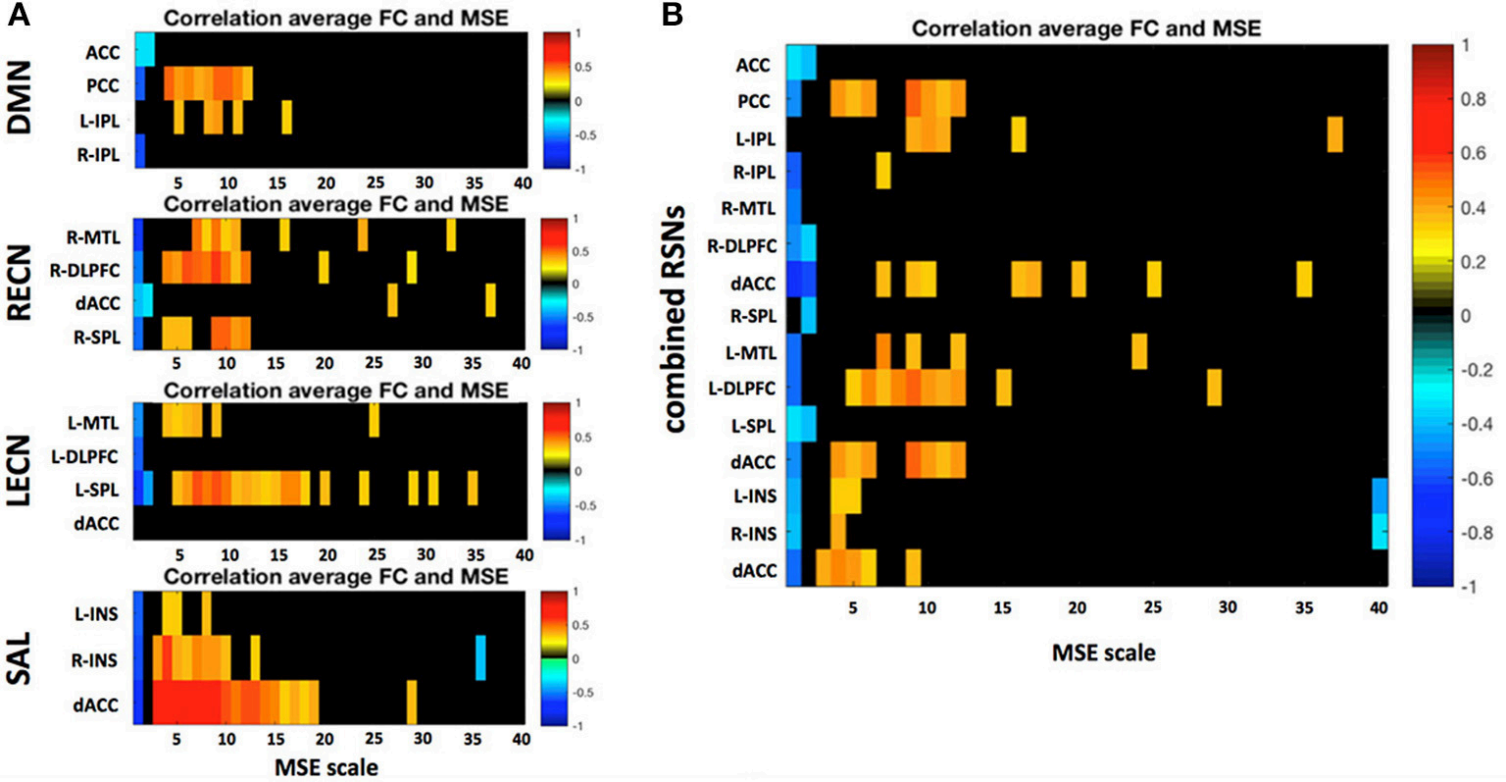
**(A)** Correlation between a region's average FC to all other nodes within the respective resting state network (RSN) and its MSE across all frequency scales. **(B)** Same analysis but all four RSNs were combined into one global network. Only significant correlations (*p* < 0.05) are depicted. Static FC shows positive correlations between MSE and FC mid to low frequencies there.

A more detailed analysis at the nodal level of the relationship between nodal MSE and the single node connectivity within the networks (average connectivity to all other nodes) revealed that the nodal signal complexity is positively correlated to average FC of a network node at low frequencies (0.020–0.087 Hz; Figure 8), similar to the pattern we observed at the network level. This finding suggest that the higher the complexity at a given node at low frequencies the more it is integrated into the network. Again this is in line with the theory that MSE at low frequencies might be related to information transfer between nodes of a network. Interestingly some nodes show very strong associations: for example the posterior cingulate cortex (PCC) in the DMN and the dorsal anterior cingulate cortex (dACC) in the SAL. We hypothesize that these areas represent hub areas in the respective networks that orchestrate the flow of information within these systems since their nodal complexity dominates the network's functional connectivity. This further indicates that complex and thus less regular signals in network nodes could allow for a more dynamic network reconfiguration and explorations of different FC states, and that network hub areas lie at the center of such reconfigurations and facilitate the information exchange between separate networks (Yang et al., 2013).

Last but not the least, conventional FC analysis often uses a band-pass filter limiting the frequency range from 0.001 to 0.1 Hz since this frequency range dominates long-range connections (Cordes et al., 2001). While higher frequencies are often contaminated by noise, there is increasing evidence that high frequency fMRI fluctuations also contribute to FC (Niazy et al., 2011; Smith et al., 2013b), especially since the advent of multiband fMRI acquisitions with sub-second temporal resolutions. Here we provide further evidence that the low-frequency fMRI fluctuations is strongly related to the long-range connectivity commonly investigated, while the MSE of high-frequency fMRI fluctuations may represent local signal processing that shows inverse correlations with FC. Our data suggest that high frequency fMRI fluctuations may also contribute to understanding the dynamic organization of brain networks in rs-fMRI (Cabral et al., 2017).

## Discussion

### Complexity analysis of brain signals

The self-similarity of neural signals across both temporal and spatial scales has been consistently observed in EEG, MEG, and fMRI studies of healthy volunteers, characterized by a power law of two-point correlation function (reviewed by Turkheimer et al., 2015). This observation indicates that a complex system such as the brain operates close to a critical point between two extreme states, one of excessive cortical integration, where long-range correlations dominate the dynamics of the system, and the other of complete segregation where activity is locally constrained. It has been suggested in brain models that operating at a point near criticality maximizes the dynamic range, sensitivity, and response time of networks to incoming information and is therefore ecologically advantageous (Beggs, 2008; de Arcangelis and Herrmann, 2010; Urban et al., 2012; Moretti and Munoz, 2013).

In electrophysiology, transient periods of synchronization of neuronal activities, typically mediated by gamma oscillations, are separated by moments of de-synchronization that mark the transition between perception and response (Rodriguez et al., 1999). Recent fMRI studies have shown that the temporal variation of FC is non-stationary with dynamic changes within time scales of seconds to minutes, and an rs-fMRI scan is characterized by frequent transitions between a repertoire of reoccurring short-term connectivity patterns termed “FC states” (Chang and Glover, 2010; Hutchison et al., 2013a,b; Allen et al., 2014). Such dynamic changes of reoccurring microstate sequences have also been observed in EEG (Van De Ville et al., 2010). There is evidence that temporal variability of FC and/or microstates is associated with behavioral performance, and may be affected by conscious and behavioral states (Thompson et al., 2013a,b; Barttfeld et al., 2015; Elton and Gao, 2015).

Based on such theory, characterizing the complexity of neural signals may indicate the brain's capacity for information processing, i.e., increased complexity of regional neural signals may indicate greater transition or exploration between different states of brain networks, thereby a greater propensity for information processing (McDonough and Nashiro, 2014; Turkheimer et al., 2015). In this sense, model-free statistical metrics such as MSE are well suited for characterizing the non-stationary dynamic changes in neural signals across multiple measurement scales.

### Relationship between neural complexity and network FC

In this paper, we investigated the relations between neural complexity (as measured by MSE) and network FC through theoretical simulations, animal models, and human rs-fMRI data. Both simulation and animal experiment showed positive correlations between MSE of regional neural signals (LFP and/or fMRI) and network FC. In human rs-fMRI, the positive association between MSE of regional fMRI signals and network FC is observed at low temporal frequencies (0.020–0.087 Hz).

The overall positive association between MSE of regional neural signals and network FC may counter the intuition that more complex neural signals interfere with phase relationships between network nodes, thereby decreasing overall FC. Nevertheless, our observation is consistent with the hypothesis that FC is mediated by a dynamic series of reoccurring “FC states” leading to an increased overall FC (averaged over a few minutes) with a greater level of regional neural complexity. Furthermore, our simulation and animal data showed a trend of decreasing associations between MSE and FC from the theta, alpha, beta, to gamma band. In rs-fMRI data, we observed positive associations between complexity and FC at low temporal frequencies (0.020–0.087 Hz), and negative correlation between complexity and FC at high temporal frequencies (0.347–0.694 Hz). This is consistent with the theory that coarse time scales or lower temporal frequencies may reflect long-range interactions across distributed neural populations, while fine time scales or higher temporal frequencies may reflect interconnectivity among local neural populations (Vakorin et al., 2011; McIntosh et al., 2014). Hence MSE at high and low temporal frequencies may represent local and distributed information processing across nodes of the network respectively.

Another observation from our study is that the overall relationship between neural complexity and network FC is replicated across measurement scales from electrophysiology to fMRI, as well as across network scales such as the whole CoCoMac and its 5 modules, the 4 major human resting brain networks and their combined whole-brain network. This observation adds to increasing evidence of scale-invariant processes observed across a number of modalities such as electrophysiology, EEG, structural and functional MRI, as well as the hypothesis on the elementary spatial brain motif underlying computations across spatially organized neuronal ensembles (Turkheimer et al., 2015). Our data may also be interpreted in the context of non-equilibrium steady state (NESS) dynamics, self-organized criticality (SOC) and slowing (Stam and de Bruin, 2004; Shin and Kim, 2006; Kitzbichler et al., 2009). Critical slowing means that some modes of SOC systems decay slowly, compared to the stable fast modes, and show protracted correlations over time which are linked with the emergence of the intrinsic brain networks. This plausible interpretation may provide a link between the underlying neural complexity and the emergent large-scale functional connectivity. Overall, our results from dynamic network modeling, animal models, and human rs-fMRI suggest that characterizing the complexity of neural signals across spatial and temporal scales provides a valuable approach, alone or in conjunction with FC, to elucidate the mechanisms of local signal processing and relation to information transfer within functionally connected brain networks.

### Potential applications of complexity analysis

We expect potential applications of brain complexity analysis in the following areas: (1) Probing the excitatory/inhibitory (E/I) balance of regional neuronal populations; (2) Characterizing conscious and behavioral states including sleep, anesthesia, and vegetative states; (3) Diagnosing neurological disorders such as epilepsy and dementia.

The complexity of local signal fluctuations has been hypothesized to be sensitive to the coordinated firing of neuronal clusters, where increased excitability causes more irregular/complex activity and increased inhibition more regular/predictable patterns (Homayoun and Moghaddam, 2007; Haider and McCormick, 2009). Hence, quantifying the complexity of dynamic brain signals using non-linear statistics such as MSE may provide a unique and innovative approach to probe the underlying neuronal fluctuations and the E/I balance. In particular, electrophysiology and fMRI recording may be combined with noninvasive neuromodulation techniques such as transcranial magnetic stimulation (TMS) and transcranial direct current stimulation (tDCS) to manipulate the regional E/I state while observing concomitant changes in neural complexity (Liang et al., 2014).

The brain operates at a point near criticality between incoherence and synchrony that facilitates its fluid transition across a repertoire of quasi-stable states. Such self-organizing capability of the brain is affected by conscious and behavioral states including sleep, anesthesia, and vegetative state. Recently, complexity analysis has been successfully applied as a consciousness test by calculating the perturbational complexity index (PCI) of TMS induced EEG response (Massimini et al., 2005; Casali et al., 2013; Casarotto et al., 2016). The PCI exploits the common video compression algorithm (e.g., MPEG) to estimate the compressibility of TMS elicited EEG response. For spontaneous neural signals over relatively long measurement periods (e.g., minutes), MSE and its variants may be more suitable for characterizing conscious and behavioral states compared to PCI that was designed for transient responses.

MSE analysis may also have clinical value for diagnosing neurological disorders such as epilepsy and dementia. Epileptic seizures are characterized by relatively large-scale synchronization of the EEG signal into high amplitude and stereotyped bursts, reflecting the recruitment of millions of neurons to fire together in a patterned manner. In other words, epilepsy is an abnormal, and toxic, self-organizing state of the brain that may benefit from complexity analysis (Engel et al., 1998). Other neurologic and psychiatric disorders such as dementia and schizophrenia are characterized by abnormal E/I balance and network FC, therefore are suitable for complexity analysis. Indeed, recent studies reported reduced entropy measures of rs-fMRI and functional near-infrared spectroscopy (fNIRS) in subjects with mild cognitive impairment and Alzheimer's disease which are correlated with cognitive performance (Wang et al., 2017; Li et al., 2018). Several of these potential applications are also showcased in this special research topic.

### Limitations and caveats of complexity analysis

Complexity analysis of electrophysiology and fMRI is still in its infancy. Several issues remain to be addressed before it can be reliably applied to basic neuroscience and clinical applications: (1) Key parameters such as pattern length *m* and matching threshold *r* have been empirically determined in existing studies. We used m of 3 and r of 0.2 for simulated data with more data points and less noise, and m of 2 and r of 0.5 for experimental data with shorter time series and higher noise. It may be possible to adaptively determine these parameters based on the estimated noise level in the data (Smith et al., 2015); (2) The test-retest repeatability of MSE analysis needs to established; and (3) Quality control and preprocessing steps of the data are also key to reliable MSE analysis. Our experience is that MSE of electrophysiology data is more reliable than that of fMRI due to higher signal-to-noise ratio (SNR) and sampling points. Nevertheless, recent development of simultaneous multislice (SMS) or multiband imaging allows high spatial and temporal resolution fMRI that is ideally suited for MSE and other complexity analysis. While community interest in complexity analysis is growing high, it can be difficult for the researchers to obtain high quality, validated, and accessible tools to perform the computationally complex analyses they require. To date, PhysioToolkit (https://physionet.org/physiotools/) (Goldberger et al., 2000) is the most comprehensive library of software for physiologic signal processing and analysis, including novel methods based on statistical physics and nonlinear dynamics (e.g., entropy), and analysis of non-equilibrium and non-stationary processes. However, PhysioToolKit was designed primarily for analyzing physiologic recordings from a single or a few channels (e.g., ECG), and therefore does not have the capability to handle high volume 4D neuroimaging data such as fMRI and EEG. Existing neuroimaging software packages such as EEGLAB, SPM, and FSL, on the other hand, lack specific modules for nonlinear complexity analysis. Our group has developed the Complexity Toolbox (http://www.fil.ion.ucl.ac.uk/spm/ext/#Complexity) as the first systematic and comprehensive software package dedicated to complexity analysis of neuroimaging data. The current version includes four metrics: Approximate Entropy (ApEn), Sample Entropy (SampEn), Multi-Scale Entropy (MSE), and Cross-ApEn for the analysis of fMRI. Further development includes wavelet based MSE and MSE of dynamic FC, as well as complexity analysis of neurophysiology data such as EEG and ECoG.

## Conclusion

Our results from theoretical modeling, animal experiment and human fMRI suggest that (1) Regional neural complexity and network FC may be two related aspects of brain's information processing: the more complex regional neural activity, the higher FC this node has with rest network nodes; (2) MSE at high and low frequencies may represent local and distributed information processing across nodes of the network. Based on literature and our data, we propose that the complexity of regional neural signals may provide an index of the brain's capacity of information processing—increased complexity may indicate greater transition or exploration between different states of brain networks, thereby a greater propensity for information processing.

## Author contributions

DW, KJ, Y-FZ, and YY contributed to the conceptualization of this paper. KJ, CF, YQ, and HL contributed data analysis. DW, KJ, Y-FZ, and YY contributed to the drafting of the manuscript.

### Conflict of interest statement

The authors declare that the research was conducted in the absence of any commercial or financial relationships that could be construed as a potential conflict of interest.
