# Correction: Neurophysiological Basis of Multi-Scale Entropy of Brain Complexity and Its Relationship With Functional Connectivity

**DOI:** 10.3389/fnins.2018.00539

**Published:** 2018-07-30

**Authors:** Danny J. J. Wang, Kay Jann, Chang Fan, Yang Qiao, Yu-Feng Zang, Hanbing Lu, Yihong Yang

**Affiliations:** ^1^Laboratory of FMRI Technology, Stevens Neuroimaging and Informatics Institute, Keck School of Medicine, University of Southern California, Los Angeles, CA, United States; ^2^Department of Psychology, Center for Cognition and Brain Disorders, Hangzhou Normal University, Hangzhou, China; ^3^Neuroimaging Research Branch, National Institute on Drug Abuse, National Institutes of Health, Baltimore, MD, United States

**Keywords:** multiscale entropy (MSE), complexity, BOLD fMRI, electrophysiology, functional connectivity (FC)

In the original article, there was an error in Acknowledgements section. We need to add an acknowledgement of Dr. Robert X. Smith for his contributions towards Figure 1.

A correction has been made to the Acknowledgements section.

This work was partially supported by the Intramural Research Program of the National Institute on Drug Abuse, the National Institutes of Health (NIH). Data from the Human Connectome Project, WU-Minn Consortium (Principal Investigators: David Van Essen and Kamil Ugurbil; 1U54MH091657) were funded by the 16 NIH Institutes and Centers that support the NIH Blueprint for Neuroscience Research; This work was also supported by NIH grant (UH2-NS100614). The authors are grateful to Drs. Michael Breakspear and Stewart Heitmann for their help with the Brain Dynamic Toolbox. The authors are also grateful to Dr. Robert X. Smith for his contribution of Figure 1.

The authors apologize for this error and state that this does not change the scientific conclusions of the article in any way.

The original article has been updated.

## Conflict of interest statement

The authors declare that the research was conducted in the absence of any commercial or financial relationships that could be construed as a potential conflict of interest.

