# Supplementary figures and images for: Neurophysiological Basis of Multi-Scale Entropy of Brain Complexity and Its Relationship With Functional Connectivity
Source: Front Neurosci. 2018 May 29;12:352. doi: 10.3389/fnins.2018.00352 (PMC5986880; doi:10.3389/fnins.2018.00352)

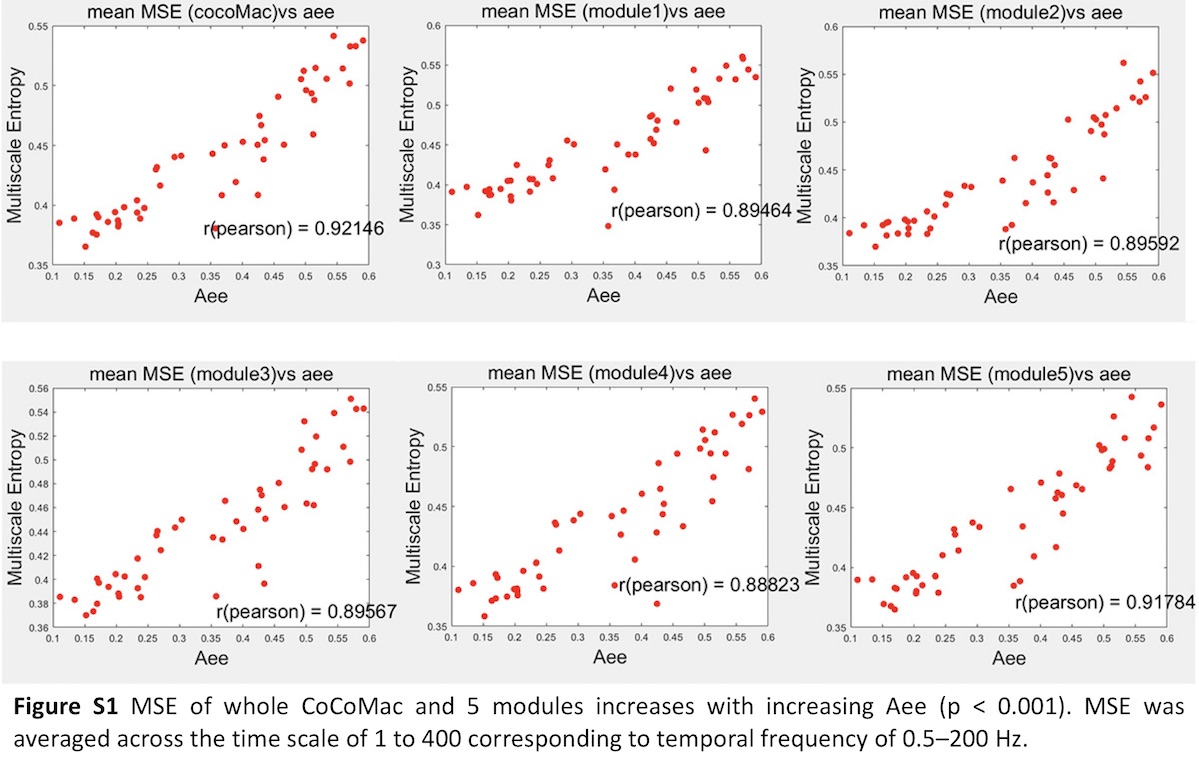

Supplement: Supplementary file 1 [file Image_1.jpg]

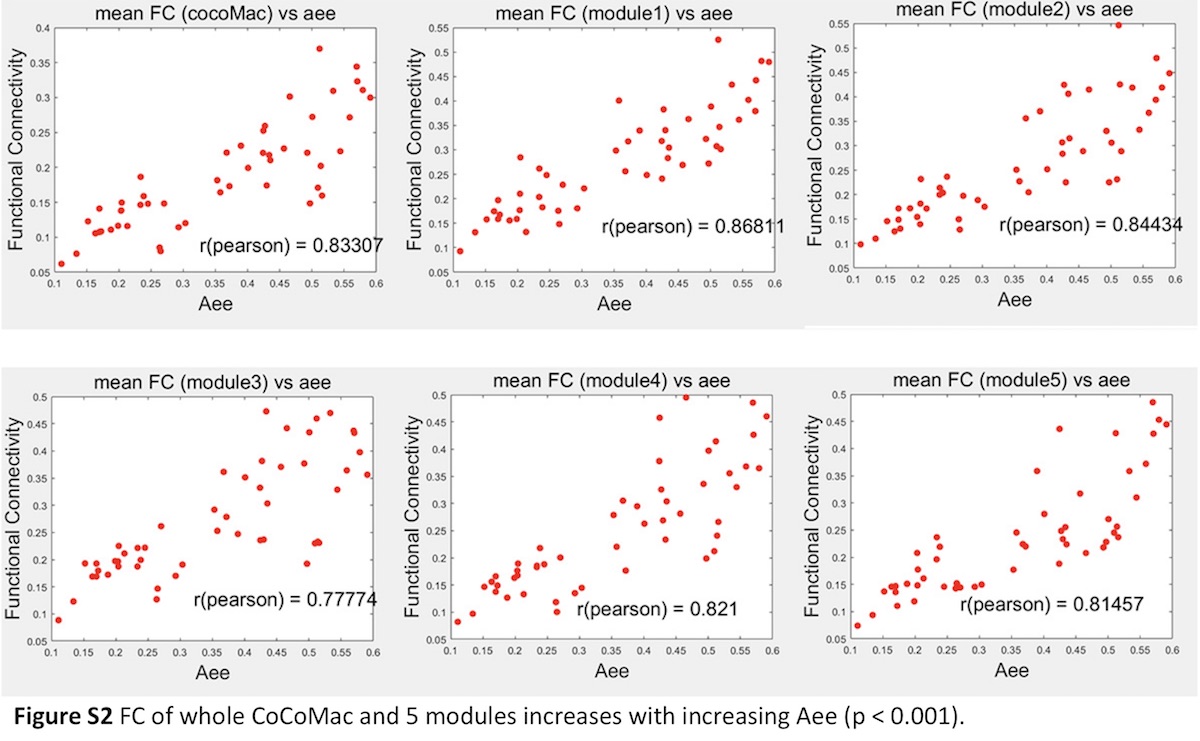

Supplement: Supplementary file 2 [file Image_2.jpg]
